# The impact of multiple supports on university students’ physical education learning motivation: a dual analysis based on SEM and fsQCA

**DOI:** 10.3389/fpsyg.2025.1446317

**Published:** 2025-03-04

**Authors:** Huaixia Hao, Qingying Zhu, Changxi Feng

**Affiliations:** ^1^School of Physical Education, Shandong University, Jinan, Shandong, China; ^2^School of Economics, University of Cologne, Cologne, Germany

**Keywords:** multiple supports, learning motivation, self-efficacy, coping style, SEM, fsQCA

## Abstract

**Background:**

During their physical education courses, university students may encounter various academic pressures and difficulties, which significantly undermine their physical education learning motivation and affect their overall development. Multiple supports from teachers, parents, and peers can effectively help students maintain confidence and enhance their physical education learning motivation. However, the underlying mechanisms by which these multiple supports influence motivation for physical education remain unclear.

**Objective:**

This study aims to explore the underlying mechanisms by which multiple supports influence learning motivation during physical education, specifically examining the mediating roles of self-efficacy and positive coping style. Additionally, it seeks to elucidate the complex configurational relationships among multiple supports, self-efficacy, coping style, and physical education learning motivation.

**Methods:**

This study employs a cross-sectional survey method to investigate Chinese university students. Through convenience sampling, 969 students were recruited from four universities in Shandong Province. The participants completed the Teacher Support Scale, Parent Support Scale, Peer Support Scale, Self-Efficacy Scale, Positive Coping Style Scale, and Learning Motivation Scale. For data analysis, statistical processing was conducted using SPSS 26.0, Amos 22.0, and fsQCA 4.1.

**Results:**

The results indicate that multiple supports from teachers, parents, and peers have significant direct effects on individual physical education learning motivation, with effect sizes of 0.132, 0.090, and 0.237, respectively. Self-efficacy acts as an independent mediator between multiple supports and physical education learning motivation, with effect sizes of 0.111, 0.076, and 0.197, respectively. Positive coping style also serve as an independent mediator in these relationships, with effect sizes of 0.091, 0.019, and 0.072; Self-efficacy and positive coping style function as a chain mediating mechanism between multiple supports and physical education learning motivation, with effect sizes of 0.021, 0.015, and 0.038; There are five equivalent configurations identified among multiple supports, self-efficacy, coping style, and physical education learning motivation.

**Conclusion:**

Support from teachers, parents, and peers not only directly influences students’ physical education learning motivation but also indirectly predicts students’ physical education learning motivation through the independent and chain mediating roles of self-efficacy and coping style. Additionally, this study elucidates the complex configurational relationships among multiple supports, self-efficacy, coping style, and physical education learning motivation, thereby validating and supplementing the results of linear analysis.

## 1 Introduction

Creating and fostering students’ motivation for physical education (PE) learning and maximizing their enthusiasm for learning is crucial for PE teaching, as its benefits extend far beyond the knowledge and skills acquired in PE classes. Numerous studies have demonstrated that the positive experiences students gain in school PE significantly influence their motivation, intentions, and attitudes toward participating in physical exercise in their daily lives (Hagger et al., 2003; Cox et al., 2008; Barkoukis et al., 2010; Standage et al., 2012). Learning motivation is a psychological process that drives individuals to engage in learning activities. It can be broadly categorized into two types: extrinsic motivation and intrinsic motivation. Extrinsic motivation primarily includes factors such as external praise, scholarships, and support from teachers and peers, which can promote learning to a certain extent (Tranquillo and Stecker, 2016). In contrast, intrinsic motivation leads to more effective learning outcomes, as it is driven by an interest in the skills and knowledge themselves rather than external rewards. When learning is achieved, the individual receives high-level feedback such as a sense of satisfaction or accomplishment (Deci et al., 1996). Additionally, learning motivation not only determines an individual’s learning outcomes but also affects their ability to learn independently. Individuals with high levels of learning motivation exhibit greater engagement in their studies, achieve better academic performance, and are more likely to engage in self-directed learning during their free time (Nabizadeh et al., 2019; Zhang and Chen, 2021; Chang and Tsai, 2022). Self-Determination Theory (SDT) further suggests that the continuous development of an individual’s motivation depends on the satisfaction of three basic psychological needs – autonomy (perceiving behavior as self-directed), competence (confidence in achieving goals), and relatedness (feeling connected to others) (Ryan and Deci, 2000). When these needs are met by teacher support, peer interaction, or institutional environment, motivation shifts from external control to intrinsic drive, significantly enhancing learning motivation. This theoretical framework constructs a key path for analyzing the motivation mechanisms of college students’ physical education: it not only focuses on the empowerment mechanisms of external support systems but also explores how these supports act as intermediaries for motivation through psychological needs.

The physical education courses referred to in this paper refer specifically to the public physical education practice courses that are compulsory for non-sports majors under the guidance of physical education teachers. These courses mainly focus on outdoor training and are designed to help university student master basic sports knowledge and sports skills and promote their physical and mental health. Essentially, the PE teaching process imparts to university students not only knowledge and skills related to physical activities but also the mindset of maintaining regular exercise. During college, students need to persist in physical learning and exercise to master lifelong applicable sports knowledge and skills, laying the foundation for future physical activity. This requires a high level of learning motivation. Current research on university students’ learning motivation primarily falls into two categories: one uses longitudinal data to uncover causal relationships (Warburton, 2017), and the other explores the influence of internal and external supports (such as teacher support, peer support, etc.) on learning motivation (Qurban et al., 2019; An et al., 2022). Although these studies have contributed to enhancing university students’ motivation for PE learning, their focus remains on the impact of individual factors on learning motivation. Therefore, further exploration of the influence of multiple supports on university students’ motivation for PE learning is urgently needed.

Based on the considerations mentioned above, it is necessary to incorporate multiple support factors and university students’ motivation for PE learning into a single model, investigating the potential relationships, influencing mechanisms, and configurational effects between them. This approach aims to provide targeted theoretical guidance for PE teaching and consequently enhance university students’ motivation for PE learning.

### 1.1 The impact of teacher support, parental support, and peer support on physical education learning motivation

Bronfenbrenner’s ecological systems theory, proposed in 1977, states that an individual’s development occurs within a multileveled, interconnected ecosystem, which includes the microsystem, mesosystem, exosystem, and macrosystem (Bronfenbrenner, 1977; Helgeson and Lopez, 2010). In the context of education, the theory emphasizes that internal factors are often the vehicle through which external factors exert their influence. For example, in a school physical education setting, external factors such as teacher support, parental support, and peer support can affect students’ internal cognitive and emotional factors, which in turn influence their motivation and behavior in physical education.

From a micro-system perspective, the academic support and emotional support of teachers in physical education directly affect individual students. Teachers’ patient guidance on students’ movements (academic support) and praise for students’ performance (emotional support) will make students feel cared for and recognized psychologically, and this positive emotional experience, as an internal factor, will enhance students’ motivation to learn physical education (Johnson et al., 1985; Patrick et al., 2007; Ruzek et al., 2016). On the contrary, students with less teacher support tend to avoid contact with teachers, leading to weak learning motivation and reduced learning efficiency (Deci and Ryan, 2000). Research by Pan and Shao (2020) also indicates a significant positive correlation between students’ learning motivation, teacher support, and learning engagement, with more attention and support from teachers correlating with increased time and effort invested by students in learning. In summary, although it is well established that teacher support back affects students’ motivation to learn, few studies have systematically investigated whether teacher support during physical education teaching still affects students’ physical education learning motivation. Therefore, it is necessary to explore the relationship between teacher support and physical education learning motivation during physical education teaching and to understand the corresponding mechanisms.

At the meso-level, parental support and the school sports teaching environment are interrelated. The material support (such as providing sports equipment) and spiritual encouragement (such as verbal praise) that parents give to their children in sports learning will form a synergistic effect with the sports teaching in school. The support that students feel in their families will make them more actively participate in school sports courses, thereby strengthening the role of teacher support in promoting students’ sports learning motivation. Previous research has indicated that parents play a significant role in determining whether children enjoy physical education learning and participation in sports (Edwardson and Gorely, 2010; Trost and Loprinzi, 2011). Parents who are passionate about sports can influence their children’s participation through both direct, practical support (providing transportation, financial support, etc.) and indirect, intangible support (verbal encouragement, emotional support, etc.), leading to stronger motivation for physical education learning and better performance (Beets et al., 2006; Pugliese and Tinsley, 2007; Edwardson and Gorely, 2010; Ryan and Deci, 2017). The relationship between parental support and adolescents’ engagement in physical education and sports has received ongoing attention (Edwardson and Gorely, 2010), and relevant systematic review studies have indicated that family involvement may enhance the effectiveness of school interventions aimed at promoting student participation in physical activities such as physical education teaching, sports competitions, leisure sports activities, etc. (Vasques et al., 2014). This suggests that the impact of parental support on individuals’ motivation for physical education learning extends beyond the family environment and may influence individuals through various channels. For university students who are undergoing the transition from adolescence to adulthood and facing the psychological impact of identity changes, as well as the need to balance academic, personal, and social aspects of life, parental support becomes even more crucial. While earlier research has predominantly focused on children, there is a paucity of studies on parental support and physical education learning among university students. Therefore, it is necessary to explore whether parental support can indeed influence the motivation for physical education learning among university students.

Peers play a crucial role in classroom learning, significantly influencing individuals’ learning motivation, adaptation to learning, and academic performance (Altermatt and Pomerantz, 2003; Kindermann, 2007; Wentzel et al., 2014). Relevant studies have found that peers can establish their unique social circles, provide emotional and behavioral support, help each other solve learning problems, accompany and engage in recreational interactions with one another (Wentzel, 1999, 2017). Perceiving high levels of peer support and encouragement, having a strong peer network, positive peer role models, and low levels of peer pressure have been shown to positively impact individuals’ learning motivation and achievement (Ryan and Patrick, 2001; Patrick et al., 2007; Wentzel, 2017). Some longitudinal research evidence also supports the role of peers in fostering individual learning motivation (Ryan and Shim, 2012; Shin and Ryan, 2014; Makara and Madjar, 2015). In fact, the dynamic changes in the establishment and development of peer relationships affect students’ performance in the learning process, with the similarity in academic goals among students and the mutual stimulation of learning motivation being closely related to peers (Shin and Ryan, 2014). For university students, peers remain an important source of ability information in the learning process (Altermatt and Pomerantz, 2003; Horn, 2004), Therefore, it is necessary to explore the potential mechanisms through which peer support stimulates physical education learning motivation among university students in the context of physical education learning.

Based on the above, this study proposes the first hypothesis:

*H1*: In the process of physical education teaching, multiple supports from PE teachers, parents and peers are positively related to university students’ physical education learning motivation.

### 1.2 The potential mediating role of self-efficacy

Self-efficacy refers to an individual’s confidence in executing plans and achieving expected goals (Bandura, 2001). Social theory suggests that self-efficacy is crucial for an individual’s learning behavior and academic performance, as it significantly influences learning motivation and emotions (Bandura, 1989). Students with high self-efficacy tend to be more confident in their academic pursuits, have stronger expectations of improving their abilities and academic achievements, thus leading them to invest more time and effort in their studies (Anam and Stracke, 2016). The Expectancy-Value Theory also points out a direct correlation between an individual’s motivation (including learning motivation) and self-efficacy (Wigfield and Eccles, 2000). Furthermore, empirical studies have indicated that self-efficacy is not only positively correlated with students’ learning motivation but also has a significant protective effect on it (Cetin-Dindar, 2016; Wu et al., 2020). Longitudinal studies have also demonstrated that self-efficacy can significantly predict an individual’s learning motivation (Alivernini and Lucidi, 2011).

The main sources influencing an individual’s self-efficacy include successful experiences, social persuasion, and emotional states. Previous research has shown that multiple supports such as from teachers, parents, and peers can effectively promote the development of individuals’ self-efficacy. Conversely, long-term lack of support may weaken students’ self-efficacy (Mercer et al., 2011). Clearly, there is a certain connection between multiple supports, self-efficacy, and learning motivation. Therefore, this study proposes the second hypothesis:

*H2*: In the process of physical education teaching, self-efficacy plays a mediating role between multiple supports and physical education learning motivation.

### 1.3 The potential mediating role of coping style

Coping style refer to the behavioral and cognitive responses individuals employ in specific situations to alleviate psychological burdens (Nattiv et al., 2007). University students may encounter various pressures and challenges during physical education learning. Adopting positive coping style (such as seeking help and changing strategies) can assist in effectively resolving issues, thereby increasing intrinsic motivation. Conversely, using negative coping style (such as avoidance, neglect, and anger) when facing challenging situations may lead to unsatisfactory outcomes (Gómez-Ortiz et al., 2016), accompanied by psychological distress (Tada, 2017). Research indicates that when students face academic difficulties, rational thinking and actively seeking help from others to obtain more resources and support can effectively solve problems, overcome learning stress, and adapt to the learning environment, thus contributing to enhanced learning motivation (Struthers et al., 2000). Therefore, positive coping style have a significant impact on maintaining learning motivation.

Multiple supports from teachers, family, and peers serve as reliable dependencies for individuals during the learning process, influencing their learning motivation, stress coping, and learning adaptation. University students who are frequently isolated and passive generally exhibit low learning motivation and are more likely to display discouragement and indecision when facing academic difficulties (Qu et al., 2023). Conversely, students under multiple supports are more willing to actively confront challenges, effectively maintaining high learning motivation, and overcoming the negative effects of stress (Hou et al., 2024). Based on the aforementioned experiences and theoretical evidence, this study proposes the third hypothesis:

*H3*: In the process of physical education teaching, coping style plays a mediating role between multiple supports and physical education learning motivation.

### 1.4 The chain mediating effect of self-efficacy and coping style

Research indicates that learning motivation may be influenced by self-efficacy and coping style (Hsiao, 2021), and all three are associated with multiple supports from teachers, parents, and peers. Coping style are also influenced by individual cognitive factors such as self-efficacy. In fact, the level of self-efficacy not only determines whether individuals can choose appropriate ways to deal with stress (Konaszewski et al., 2021), but also represents their confidence in overcoming difficulties (Bandura, 1977). Therefore, when facing academic difficulties or other stressful situations, students with high self-efficacy tend to choose positive coping style to solve problems and strive to cope with adverse situations, while students with low self-efficacy may give up prematurely or choose to avoid difficulties (Bandura and Locke, 2003; Geng et al., 2018; Chen et al., 2020; Liu et al., 2020).

Recent relevant studies have identified the potential mechanism between external factors and learning motivation, confirming that self-efficacy and positive coping style play a mediating role in this mechanism (Liu and Li, 2019). Furthermore, multiple supports from various sources are important predictors of self-efficacy and coping style. Based on the research evidence mentioned above, this study posits that there may exist an indirect effect path of multiple supports → self-efficacy → positive coping style → physical education learning motivation between multiple supports and physical education learning motivation. Therefore, this study proposes the fourth hypothesis:

*H4*: In the process of physical education teaching, Self-efficacy and coping style play a chain mediating role between multiple supports and physical education learning motivation.

**Figure 1:**
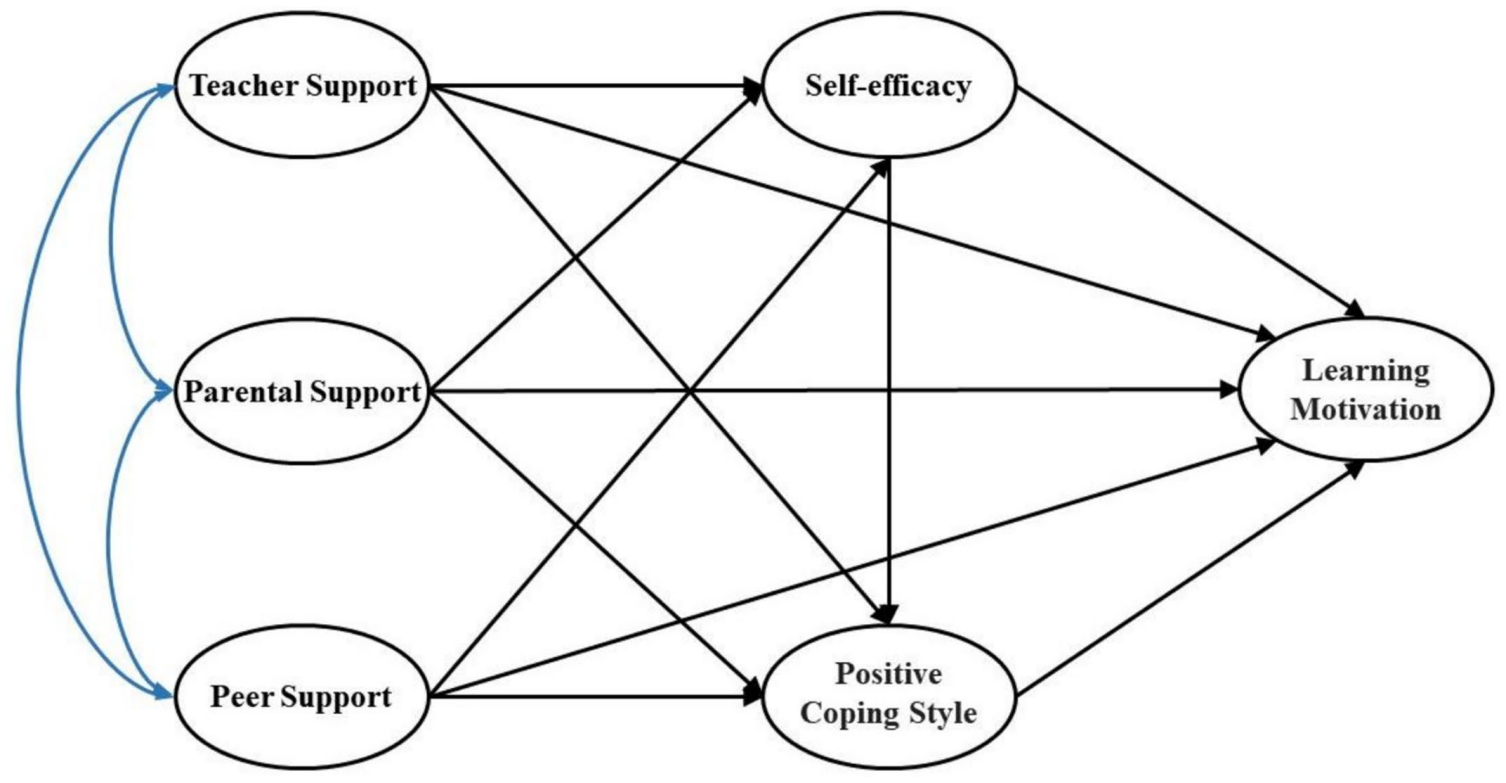
Hypothetical model.

In summary, this study focuses on Chinese university students and aims to explore the relationship between multiple supports from PE teachers, parents, peers and physical education learning motivation during public physical education practice courses. It also investigates whether self-efficacy and coping style mediate this relationship. Additionally, the study will examine the complex configuration of multiple supports from PE teachers, parents, and peers, along with self-efficacy, coping style and physical education learning motivation. This research not only helps expand the factors influencing university students’ motivation in physical education but also provides theoretical foundations for interventions and plans aimed at improving university students’ motivation in physical education. Figure 1 illustrates the hypothetical model of this study.

## 2 Materials and methods

### 2.1 Participants and data collection

This study adopted convenience sampling method, considering the limitations of time and resources in the actual research process, and selected 4 universities in Shandong Province using the convenience sampling method. It should be noted that in order to maximize the representativeness of the sample under the existing conditions, we conducted random sampling on the basis of convenience sampling. The specific operation was to randomly select students to participate in the survey from the selected 30 classes using a random number table. The research objects were all full-time undergraduate students from the first year to the third year of university, with ages mainly concentrated between 18 and 22 years old. Since the physical education courses have already ended in the fourth year of university, fourth-year university students were no longer included in the research sample. These students were all participating in the compulsory public physical education practice courses for non-physical education major students, such as basketball, volleyball, martial arts, aerobics, and so on, and were willing to participate in this study.

Initially, this study established exclusion criteria for invalid questionnaires: Firstly, incomplete provision of personal basic information. Secondly, selection of options in questions lacking logical coherence, displaying clear logical errors. Thirdly, more than 80% of responses being identical, indicating a pattern of consistent answering. Fourthly, more than 10 instances of missing answers throughout the entire survey. Fifthly, responses not meeting the specified requirements, demonstrating a phenomenon of answering questions that were not asked. Sixthly, selecting two or more options for single-choice questions. Subsequently, questionnaires were distributed to 1,050 students across 30 classes, and after screening based on the exclusion criteria for invalid questionnaires, 969 valid questionnaire samples were obtained, resulting in an effective response rate of 92.3%.

Respondents were all adults (18 years and older), aged 20.6 ± 0.78 (M ± SD); there were 549 males (56.66%) and 420 females (43.34%); 557 were only children (57.48%), and 412 were not (42.52%). The distribution by academic year was as follows: Freshman 334 (34.47%), sophomore 307 (31.68%), and junior 328 (33.85%) (Table 1).

**Table 1:** Descriptive statistical analysis.

Variables	Categories	Frequency	Percentage (%)
Gender	Male	549	56.66
	Female	420	43.34
Only/Non-only Children	Yes	557	57.48
	No	412	42.52
Grade	Freshman	334	34.47
	Sophomore	307	31.68
	Junior	328	33.85

### 2.2 Instruments

The formal questionnaire of this study comprises two parts. The first part focuses on gathering basic demographic information. The second part incorporates comprehensive scales to assess various variables involved in the study.

#### 2.2.1 Teacher support

The Teacher Support Scale, adapted for the purposes of this study, was utilized to assess the perceived level of teacher support among university students (Ober et al., 2021; Hagger et al., 2007). This scale comprises 10 items covering academic support (e.g., “During physical education classes, the teacher is willing to guide your movements”) and emotional support (e.g., “During physical education classes, your teacher often praises your performance”), utilizing a 5-point Likert scale (1 = Strongly Disagree, 5 = Strongly Agree). Higher scores indicate a greater level of perceived teacher support. The Cronbach’s *α* coefficient for this scale in our study was 0.867.

#### 2.2.2 Parental support

Parental support was assessed using the family subscale of the Multidimensional Scale of Perceived Social Support (MSPS), appropriately adapted for the purposes of the study (Zimet et al., 1988; Hagger et al., 2007), replace “family” with “parents” (for example, I can discuss recent difficulties encountered in physical education teaching with my own parents). This subscale has 4 items in total and uses a 5-point Likert scoring method (1 = strongly disagree, 5 = strongly agree). The higher the score, the more parental support the individual receives. The Cronbach’s *α* of this scale in this study was 0.815.

#### 2.2.3 Peer support

Peer support was measured using the Sports Social Support Scale compiled by Sallis et al. (1987) and adapted by Chen et al. (2017) and Hsu et al. (2011). This scale has 5 items in total (for example, your peers often encourage you to complete assignments and plans related to physical education classes), using a 5-point Likert scoring method (1 = strongly disagree, 5 = strongly agree), the higher the score, the higher the score. High indicates that the individual receives more peer support. The Cronbach’s *α* of this scale in this study was 0.825.

#### 2.2.4 Self-efficacy

The Chinese version of general self-efficacy developed by Schwarzer et al. (1997) was used to measure individual self-efficacy levels. This scale has 10 items in total (e.g., I can always solve problems if I try my best), using a 5-point Likert scale (1 = strongly disagree, 5 = strongly agree), the higher the score, the indicates that the individual’s self-efficacy is stronger. The Cronbach’s α of this scale in this study was 0.863.

#### 2.2.5 Active coping style

The active coping style subscale of the simplified coping style scale compiled by Lazarus and Folkman (1987) was used to measure the individual’s active coping style. Xie (1998) have demonstrated that this scale has good reliability and validity in a Chinese environment. This scale has 10 items in total and adopts a 5-point Likert scoring method (1 = never, 5 = often). The higher the score on the active coping dimension, the greater the individual’s tendency to adopt active coping style. The Cronbach’s *α* of this scale in this study was 0.905.

#### 2.2.6 Physical education learning motivation

The learning motivation scale compiled by Chi and Xin (2006) was used and adapted appropriately according to the purpose of this study to measure the individual’s physical education learning motivation. This scale has a total of 30 items, including two dimensions: extrinsic motivation and intrinsic motivation, and adopts a 5-point Likert scoring method (1 = completely disagree, 5 = completely agree). The higher the score, the stronger the individual’s learning motivation. The Cronbach’s *α* of this scale in this study was 0.869.

### 2.3 Data analysis

The data analysis for this study was conducted using SPSS 26.0, Amos 22.0, and fsQCA 4.1. Firstly, Harman single-factor analysis was performed using SPSS to detect any significant common method bias. Secondly, Pearson correlation analysis was used to explore the relationships among multiple support, self-efficacy, coping style, and learning motivation. Thirdly, SEM (Structural Equation Modeling) was employed through Amos to investigate the direct and mediating effects among variables in depth. Finally, fsQCA 4.1 software was utilized for fuzzy-set qualitative comparative analysis. This method, which assumes asymmetric relationships between dependent and independent variables, enables the exploration of complex non-linear causal relationships. According to Afonso et al.’s (2018) suggestion, level 5 is set as complete membership, level 1 is set as complete non-membership, and level 3 is set as the crossover point for data calibration. This is followed by a necessity analysis. Finally, a sufficiency analysis is performed to generate different configurations of mutual combinations of antecedent variables.

The rationale for conducting fsQCA analysis on top of SEM analysis in this study is as follows: (1) Using SEM and other traditional statistical methods to handle symmetric relationships, then applying fsQCA to explain causal asymmetry, can further reveal the effects of various variables on physical education learning motivation. (2) fsQCA can comprehensively analyze necessity and sufficiency relationships, effectively avoiding negative effects caused by multicollinearity. Furthermore, it helps to elucidate the complex causal interactions among multiple support, self-efficacy, coping style, and physical education learning motivation. (3) After identifying the interrelationships among variables using SEM, utilizing fsQCA based on set theory can further derive multiple equivalent strategies for enhancing students’ motivation in physical education.

### 2.4 Common method bias test

Due to the self-reported nature of data collection in this study, there is a potential for common method bias. To address this issue, the Harman single-factor test was employed. Principal component analysis was conducted on all measurement items without rotation. The results showed that the first principal component explained variance was 16.33%, which is below the critical threshold of 40%. Therefore, this study does not exhibit significant common method bias, allowing for subsequent statistical analysis.

## 3 Results and analyses

### 3.1 Correlation analysis

The study employed Pearson correlation analysis to assess the relationships between variables. The results are presented in Table 2. Significant positive correlations were found between all pairs of variables. This provides preliminary evidence supporting the validity of the hypotheses proposed in this study. Further investigation and analysis of these findings are warranted.

**Table 2:** Matrix of correlation coefficients of the study variables.

Variate	TES	PAS	PES	Se	PCS	LM
TES	1					
PAS	0.199**	1				
PES	0.440**	0.213*	1			
Se	0.555**	0.253**	0.571**	1		
PCS	0.464**	0.282**	0.574**	0.609**	1	
LM	0.465**	0.274**	0.549**	0.581**	0.628**	1

TES is Teacher Support; PAS is Parental Support; TES is Peer Support; Se is Self-efficacy; PCS is Positive Coping Style; LM is Learning Motivation; *: significant at 0.05 level; **: significant at 0.01 level.

### 3.2 SEM results

**Figure 2:**
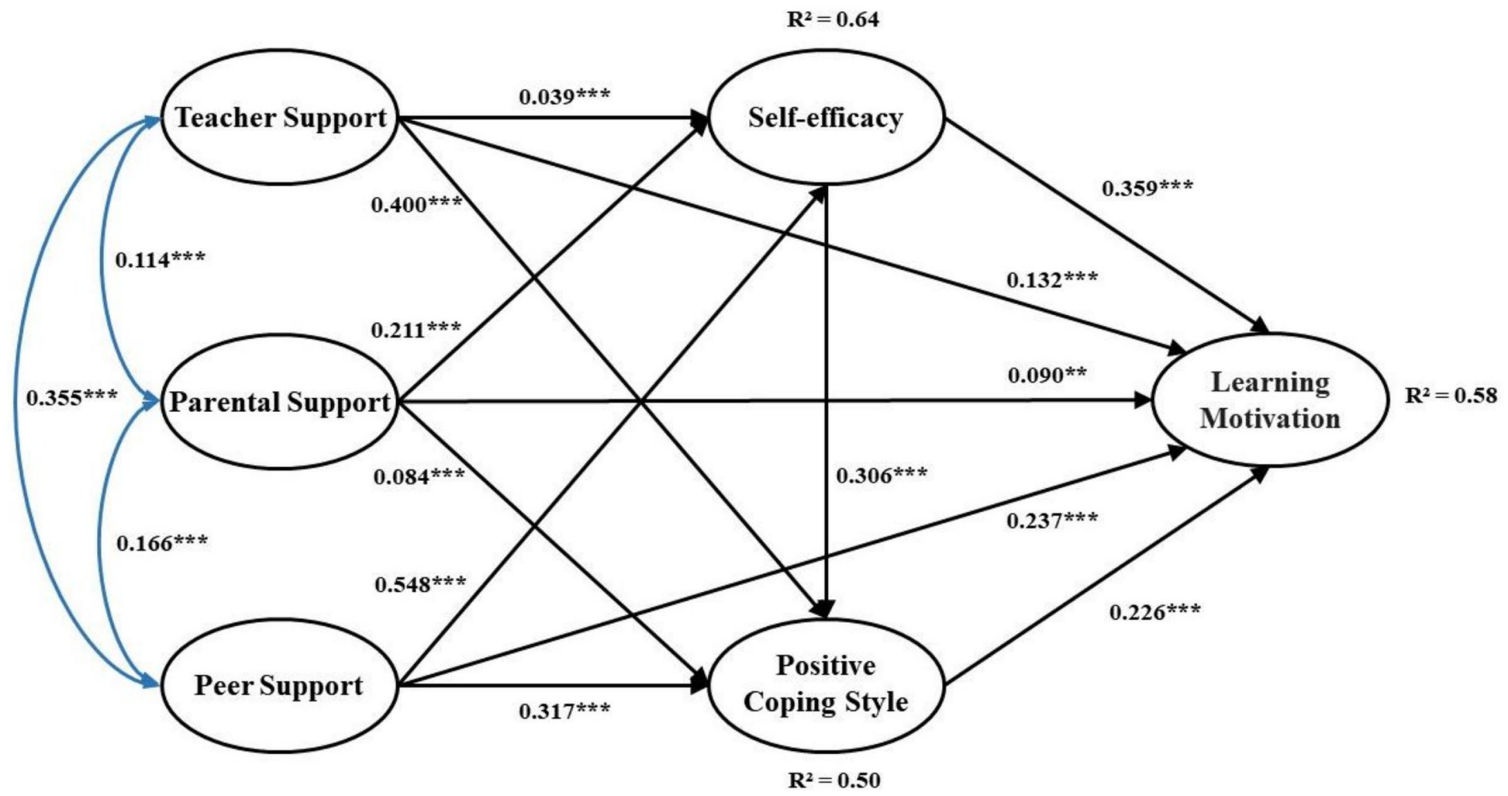
Model path diagram. **significant at 0.05 level; ***significant at 0.01 level.

After completing the correlation analysis, the next step is to examine the structural equation model. Firstly, the model fit is good: *χ^2^/df* = 1.944; GFI = 0.936; NFI = 0.946; IFI = 0.973; CFI = 0.973; RMSEA = 0.043. Secondly, further analysis was conducted to investigate the direct impact relationships between variables. As shown in Table 3 and Figure 2, teacher support has a significant positive effect on physical education learning motivation (*β* = 0.09, *p* < 0.01); parental support has a significant positive effect on physical education learning motivation (*β* = 0.065, *p* < 0.01); peer support has a significant positive effect on physical education learning motivation (*β* = 0.205, *p* < 0.01). Therefore, in the process of physical education teaching, multiple supports from teachers, parents and peers are positively related to university students’ physical education learning motivation. Hypothesis 1 is supported. Additionally, this study also examined the direct impact relationships between other variables. The results show that all direct impact paths related to the study are positively significant. The next step is to test the possible mediating relationships between variables.

**Table 3:** Test results for direct impact.

Path	Effect size	*p*-value
TES → LM	0.132	***
PAS → LM	0.090	0.023
PES → LM	0.237	***
TES → Se	0.039	***
TES → PCS	0.400	***
PAS → Se	0.211	***
PAS → PCS	0.084	***
PES → Se	0.548	***
PES → PCS	0.317	***
Se → LM	0.359	***
Se → PCS	0.306	***
PCS → LM	0.226	***

***Significant at 0.01 level.

The Bootstrap method was used to resample the sample 2000 times with a confidence interval set at 95%CI to test the significance of the mediating effects specified in the model. The results of the mediating effect test are shown in Table 4. Firstly, the explained variances of self-efficacy, coping style, and physical education learning motivation were 64, 50, and 58%, respectively, indicating that these explained variances have a high predictive ability. Secondly, observing specific path effects, it was found that self-efficacy and positive coping style, respectively, mediate the relationship between teacher support, parental support, peer support, and physical education learning motivation. Therefore, in the process of physical education teaching, self-efficacy and coping style mediate the relationship between multiple supports and physical education learning motivation. Hypotheses 2 and 3 are supported. Lastly, the study validated three specific chain-mediated paths: TES → Se → PCS → LM, PAS → Se → PCS → LM, and PES → Se → PCS → LM. Hypothesis 4 is supported.

**Table 4:** Results of the mediation effect test.

	Effect	Path	Effect size	Bootstrap 95%CI		Effect ratio
				Lower	Upper	
Model 1	Total	TES → LM	0.355	0.233	0.472	
	Direct	TSE → LM	0.132	0.023	0.246	37.18%
	Ind 1	TES → Se → LM	0.111	0.061	0.170	31.27%
	Ind 2	TES → PCS → LM	0.091	0.036	0.155	25.64%
	Ind 3	TES → Se → PCS → LM	0.021	0.008	0.040	5.91%
Model 2	Total	PAS → LM	0.200	0.107	0.302	
	Direct	PAS → LM	0.090	0.002	0.186	45.00%
	Ind 4	PAS → Se → LM	0.076	0.043	0.121	38.00%
	Ind 5	PAS → PCS → LM	0.019	0.003	0.048	9.50%
	Ind 6	PAS → Se → PCS → LM	0.015	0.006	0.030	7.50%
Model 3	Total	PES → LM	0.544	0.452	0.635	
	Direct	PES → LM	0.237	0.134	0.336	43.57%
	Ind 7	PES → Se → LM	0.197	0.139	0.263	36.21%
	Ind 8	PES → PCS → LM	0.072	0.029	0.120	13.24%
	Ind 9	PES → Se → PCS → LM	0.038	0.016	0.066	6.98%

### 3.3 fsQCA results

#### 3.3.1 Calibrations

The data was calibrated so that the membership scores for each variable fell between 0 and 1, facilitating statistical analysis of configurational consistency and coverage. Since all scales used in this study were based on a Likert five-point scale, a direct calibration method was employed. Level 5 is set as complete membership, level 1 is set as complete non-membership, and level 3 is set as the crossover point for calibration analysis.

#### 3.3.2 Necessary condition analysis

According to Ragin’s (2008) recommendation, a condition variable is considered a necessary condition that can independently explain the outcome variable when its consistency level exceeds 0.9. The analysis results, as shown in Table 5, reveal that when physical education learning motivation (LM) is taken as the outcome variable, only self-efficacy (Se) qualifies as a necessary condition for enhancing physical education learning motivation; the remaining condition variables are not necessary conditions. However, the consistency levels of most condition variables are very close to 0.9, indicating the reasonableness of the earlier variable selection and hypotheses. When non-physical education learning motivation (~Se) is the outcome variable, all condition variables exhibit consistency levels below 0.9, signifying that these conditions are not independent necessary conditions for explaining non-physical education learning motivation. Additionally, the majority of condition variables have coverage values exceeding 0.6, suggesting a certain degree of influence on the outcome variable. Consequently, the next step involves exploring the configurations generated by different combinations of condition variables.

**Table 5:** Result of necessary condition analysis.

	LM	
	Consistency	Coverage
TES	0.846	0.840
TES~	0.426	0.728
PAS	0.824	0.803
PAS~	0.438	0.771
PES	0.861	0.848
PES~	0.395	0.683
Se	0.902	0.852
Se~	0.341	0.637
PCS	0.881	0.845
PCS~	0.364	0.660

	~LM	
	Consistency	Coverage
TES	0.732	0.431
TES~	0.728	0.738
PAS	0.781	0.452
PAS~	0.660	0.690
PES	0.691	0.404
PES~	0.739	0.759
Se	0.673	0.377
Se~	0.738	0.817
PCS	0.684	0.390
PCS~	0.728	0.784

#### 3.3.3 Sufficiency analysis

Combining previous research and considering the specific circumstances of this study, we set the consistency threshold at 0.8, PRI threshold at 0.7, and frequency threshold at 1 to construct the truth table, following standards from Fiss (2011) and Thiem (2017). This process yielded complex solutions, intermediate solutions, and simple solutions. Given that the complex solutions and intermediate solutions were consistent, this study primarily focuses on intermediate solutions, with simple solutions as supplementary, to identify core and marginal conditions. The configuration analysis results, as shown in Table 6, revealed three key findings: (1) LM1, LM2, and LM3 configurations correspond to three unique chain-mediated pathways identified in the SEM analysis, thus validating each other and enhancing the credibility of the research findings. (2) The LM1, LM2, and LM3 configurations also indicate that when self-efficacy and positive coping style simultaneously appear as core conditions, meeting any of teacher support, parental support, or peer support is sufficient to achieve a significant mechanism for high physical education learning motivation. (3) The LM4 and LM5 configurations demonstrate that when teacher support, parental support, and peer support all appear as core conditions, meeting either self-efficacy or positive coping style is adequate to achieve a significant mechanism for high physical education learning motivation.

**Table 6:** Result of configuration analysis.

Configuration	LM1	LM2	LM3	LM4	LM5
TES	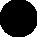			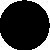	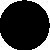
PAS		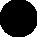		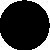	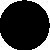
PES			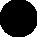	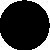	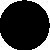
Se	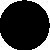	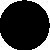	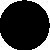	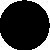	
PCS	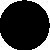	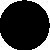	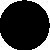		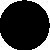
Consistency	0.924	0.920	0.928	0.933	0.934
Raw coverage	0.751	0.726	0.767	0.663	0.667
Unique coverage	0.013	0.009	0.020	0.018	0.023
Overall solution consistency	0.856				
Overall solution coverage	0.862				

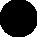
 represents the edge existence condition; 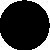 represents the core existence condition; The blank area represents whether the condition is present or missing.

## 4 Discussion

This study explored the relationships between perceived multiple support, self-efficacy, coping style, and physical education learning motivation among university students during physical education instruction. The results indicated that multiple support not only directly influences students’ motivation for physical education learning but also exerts an indirect effect through self-efficacy and positive coping style. Additionally, there exists a complex non-linear configurational relationship among multiple support, self-efficacy, positive coping style, and physical education learning motivation. This section will summarize these findings and discuss them in conjunction with existing literature.

First, the findings of this study indicate that multiple support from PE teachers, parents, and peers positively influences university students’ motivation for physical education learning. This is consistent with the results of Ruzek et al. (2016), Warburton (2017), and Qurban et al. (2019), who found a significant positive association between multiple support from PE teachers, parents, and peers and university students’ motivation for physical education learning. Timely academic and emotional feedback from teachers and peers can directly or indirectly enhance students’ willingness to engage in learning activities (Nelson and DeBacker, 2008; Ruzek et al., 2016; Sadoughi and Hejazi, 2021; Jane, 2014). Notably, this study found that peer support (*β* = 0.237) had a greater impact on individual physical education learning motivation than teacher support (*β* = 0.132). This can be attributed to the fact that university students, unlike middle or high school students, spend less time interacting with teachers and more time living with their peers, leading to a greater influence from peer support. Additionally, parental support is also a significant factor in enhancing individual motivation for physical education learning. Research has shown that parental support is a crucial factor influencing the intention of adolescents, including university students, to engage in physical exercise (Fredricks and Eccles, 2004). Classic studies have demonstrated that parent–child interactions shape their initial motivation and values toward participating in physical activities (Harter, 1982; Bandura, 1986). Recent studies have validated that both tangible and intangible support provided by parents can significantly enhance students’ motivation for participation in physical education learning (Qurban et al., 2019). In summary, multiple support from PE teachers, parents, and peers positively influences university students’ motivation for physical education learning in various ways.

Second, the results of this study indicate that self-efficacy plays a mediating role between multiple support and physical education learning motivation. Perceived multiple support not only directly predicts students’ physical education learning motivation but also indirectly influences it through self-efficacy. According to self-determination theory, every individual has three psychological needs: autonomy, relatedness, and competence. When these basic psychological needs are met, both intrinsic and extrinsic motivation are enhanced (Deci and Ryan, 2000). The supportive behaviors of teachers, parents, and peers, along with heightened self-efficacy, fulfill students’ needs for autonomy, relatedness, and competence, thereby enhancing their physical education learning motivation (Jin and Wang, 2019; Tang and He, 2023). Previous studies have also demonstrated that when students receive support from teachers, parents, and peers, their self-efficacy significantly increases, leading to greater focus on classroom learning (Tang and He, 2023). Specifically, the more positive support students receive from various sources, the higher their learning motivation is likely to be. Therefore, in the context of physical education, teachers should play a primary role in creating a positive teaching atmosphere by providing more guidance and emotional support. Additionally, they should help students establish good peer relationships and encourage parents to communicate with their children, fulfilling students’ basic psychological needs. This approach can stimulate strong motivation and intention for learning in physical education.

Third, coping style serve as a crucial mediating variable in the complex relationship between multiple support and physical education learning motivation. This study finds that support from teachers, parents, and peers significantly positively influences students’ motivation to learn in physical education. And, this influence is also mediated by positive coping style. This suggests that multiple supports from various sources are more conducive to the development of positive coping styles and mindsets among students, making them more willing to find more effective learning solutions to overcome current discomforts in the face of academic difficulties and setbacks (Hou et al., 2024). In contrast, students who perceive low levels of support often adopt negative coping style, tending to avoid challenges in life or academics (Cherkil et al., 2013). This avoidance leads to the accumulation of negative emotions, a significant decrease in learning motivation, and ultimately, a sense of aversion to studying (Qu et al., 2023). Furthermore, consistently using negative coping style and accumulating negative emotions can result in conflicts and confrontations with teachers and peers (Henderson et al., 2003), severely impacting student-teacher and peer relationships, and consequently decreasing learning motivation (Shen et al., 2021). To sum up, in the process of physical education learning, active coping style is particularly important. It plays a mediating role between multiple supports and physical education learning motivation.

Fourth, the results of this study indicate that self-efficacy has a significant positive impact on positive coping style (Jex et al., 2001; Konaszewski et al., 2021). Furthermore, self-efficacy and positive coping style act as a chain mediator between multiple sources of support and learning motivation in physical education. During the learning process of physical education courses, it is inevitable for some university students to encounter academic challenges and pressure. Research has shown that students with higher self-efficacy are more likely to adopt positive coping style to learn and find other ways to handle stressful events and problems, thereby possessing stronger learning motivation (Ma et al., 2022). Other studies also suggest that multiple sources of support from teachers, parents, and peers influence individuals’ self-efficacy, encouraging them to handle difficulties more actively and thereby enhancing their learning motivation (Struthers et al., 2000). This study confirms these findings. In summary, multiple support can significantly predict the level of self-efficacy, and self-efficacy can influence physical education learning motivation through positive coping style.

Fifth, previous research has primarily focused on identifying the symmetrical relationships among multiple sources of support, self-efficacy, coping style, and learning motivation. In contrast, this study employs fsQCA analysis, providing an in-depth interpretation of the asymmetrical and complex interrelationships between these variables. By demonstrating that the combination of multiple support, self-efficacy, and coping style can enhance students’ motivation for physical education, this study introduces complexity theory and methodology into the field of sports education research for the first time (Woodside, 2014; Pappas, 2019; De et al., 2020). Moreover, it reveals alternative configurations of multiple support, self-efficacy, and coping style that influence students’ motivation for physical education. These configurations can explain equifinality, verifying and complementing the results of linear analyses.

## 5 Research limitations and future directions

Although this study provides a reference for improving students’ learning motivation in the process of physical education teaching, there are still certain limitations, and future research will make relevant expansions. First, this study focuses on Chinese university students, examining the relationships between multiple support, self-efficacy, coping style, and physical education learning motivation. Future research could adopt different methodologies and expand sample sizes to include more diverse populations and cultural contexts, thereby testing the reliability and generalizability of the findings. Second, while this study identifies self-efficacy and coping style as mediating variables, they represent only a fraction of potential mediators. Future research should aim to identify and test other potential mediating variables, which would significantly enrich the current findings. Third, due to the limited conditions for conducting research, a convenient sampling method was used for the cross-sectional questionnaire survey, so this study did not provide evidence for the time sequence or causal relationship. Therefore, the results of this study should be generalized with caution. Future research can find a batch of specific experimental subjects and overcome this limitation through longitudinal survey experiments.

## 6 Conclusion

The findings indicate that support from PE teachers, parents, and peers can directly influence students’ physical education learning motivation. Furthermore, these sources of support can indirectly predict students’ physical education learning motivation through the independent and chain mediating effects of self-efficacy and coping style. Additionally, this study has clarified the complex configurational relationships among multiple support, self-efficacy, coping style, and learning motivation, thereby verifying and complementing the results of linear analyses.

To enhance students’ learning motivation in physical education, it is essential to consider positive support from various sources, prove individual self-efficacy, and encourage students to adopt proactive measures to solve problems. This approach not only fosters students’ enthusiasm for learning but also helps in developing their positive, optimistic, and autonomous characteristics, which are beneficial for their sustainable development.

## Data Availability

The original contributions presented in the study are included in the article/supplementary material, further inquiries can be directed to the corresponding author.
